# Disseminated herpes zoster following tear gas exposure in a patient on upadacitinib

**DOI:** 10.1016/j.jdcr.2026.04.072

**Published:** 2026-05-20

**Authors:** John Sauer, Kathleen Jedruszczuk, Adriana Ros

**Affiliations:** a Rowan-Virtua School of Osteopathic Medicine, Stratford, New Jersey; b Department of Dermatology, Hackensack Meridian Health, Palisades Medical Center, North Bergen, New Jersey; c Dermatology Institute and Laser Center, Rockaway, New Jersey

**Keywords:** atopic dermatitis, CS gas exposure, disseminated herpes zoster, immunosuppression, JAK inhibitor, tear gas, upadacitinib, varicella-zoster virus

## Introduction

Herpes zoster results from reactivation of varicella-zoster virus (VZV) and commonly affects older adults, typically with a history of primary VZV infection.^1^ The classic clinical presentation consists of grouped clusters of vesicles on an erythematous base distributed along a unilateral dermatome and is often preceded by neuropathic symptoms.^1^^,^^2^ Disseminated herpes zoster occurs predominantly in immunocompromised individuals. Impairment in cell-mediated immunity, whether due to systemic illness or immunocompromised states, is a primary factor contributing to disseminated reactivation.^2^^,^^3^ Disseminated herpes zoster is defined as >20 vesicular lesions outside the primary and adjacent dermatomes and carries an increased risk of complications such as pneumonitis, hepatitis, and meningoencephalitis.^2^, ^3^, ^4^

Janus kinase (JAK) inhibitors are increasingly used in dermatology for the management of inflammatory and autoimmune conditions. Currently, oral JAK inhibitors are Food and Drug Administration–approved for the treatment of moderate-to-severe atopic dermatitis (AD) and severe alopecia areata, and a topical formulation is approved for both AD and nonsegmental vitiligo. In addition, delgocitinib was recently the first Food and Drug Administration–approved, steroid-free, topical pan-JAK inhibitor cream indicated for moderate-to-severe chronic hand eczema. JAK inhibitors increase infection risk, particularly herpes zoster, through inhibition of antiviral cytokine pathways such as interferon-γ and interleukins (ILs) 2 and 15.^3^^,^^5^^,^^6^ While these mechanisms provide therapeutic benefit in various inflammatory dermatoses, they may increase susceptibility to VZV reactivation. Here, we present the first documented case of disseminated herpes zoster occurring after tear gas exposure in a 33-year-old male receiving upadacitinib for AD.

## Case report

A 33-year-old male with a medical history of AD, managed with upadacitinib 30 mg once daily for several years, presented with a 4-day history of a blistering and ulcerative papular rash. Eight days prior to presentation, he was exposed to tear gas, CS gas (2-chlorobenzalmalononitrile), and pepper spray during police academy training. After exposure, he reported ocular symptoms, including burning, visual disturbances, and swelling of the eyelids.

Four days after exposure, the patient developed blistering lesions on the left posterior arm, which progressed to involve the face, trunk, and bilateral upper and lower extremities. The rash was mildly pruritic and painful. The patient denied fevers, headaches, and numbness or tingling. This progression of symptoms prompted him to visit the emergency department, where he was treated for presumed tear gas–related dermatitis with oral prednisone (20 mg twice daily), hydrocortisone (2.5%) lotion, and analgesics. He reported minimal improvement of his symptoms at that time, however noted previously formed blisters started to scab.

**Fig 1:**
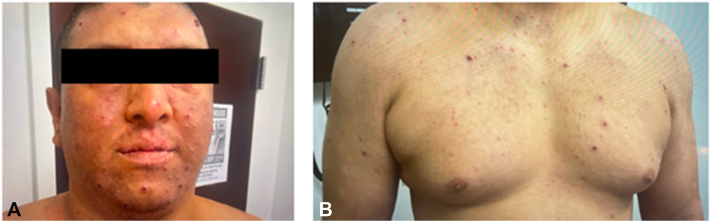
**A** and **B,** The face and chest reveal numerous scattered 2 mm to 1 cm eroded vesicles and grouped papules with an erythematous base.

**Fig 2:**
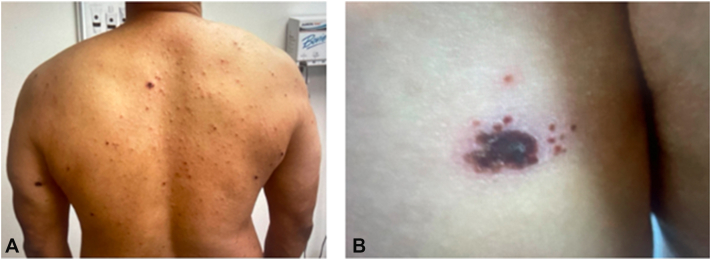
**A** and **B,** The back and left arm reveal numerous scattered 2 mm to 1 cm eroded vesicles and grouped papules with an erythematous base.

**Fig 3:**
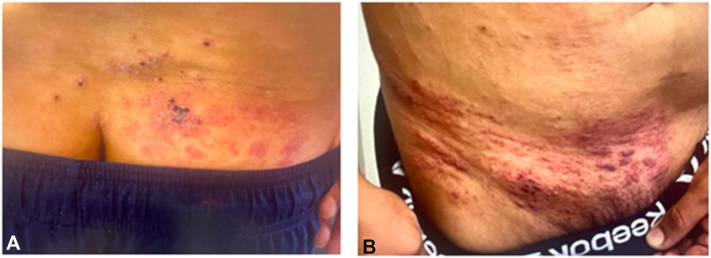
**A** and **B,** The right gluteal region reveals erythematous papulovesicles and plaques with central erosions extending in a dermatomal pattern to the right suprapubic region.

Physical examination revealed numerous scattered eroded vesicles and grouped papules with an erythematous base throughout the face, trunk, and bilateral upper and lower extremities (Figs 1 and 2). On the right gluteal region, there were erythematous papulovesicles and plaques with central erosions extending in a dermatomal pattern to the right suprapubic region (Fig 3).

Given the concern for chemical burns secondary to CS gas exposure, poison control was contacted. Toxicology recommendations included transferring the patient to a burn unit, intravenous antibiotics, topical antibiotics, and administration of tetanus vaccination if not up to date. During hospitalization, a viral swab was obtained from a vesicle in the gluteal region which resulted in a positive VZV polymerase chain reaction. Given the presence of more than 20 vesicles outside of the primary and adjacent dermatomes, the patient was diagnosed with disseminated herpes zoster. Upadacitinib was discontinued and antiviral treatment with acyclovir was initiated. The patient was discharged and instructed to follow up with dermatology within 1 week for reevaluation.

## Discussion

This case emphasizes the diagnostic complexity of occupational chemical exposure in an immunomodulated patient. The patient’s exposure to CS gas during police academy training raised concern for irritant contact dermatitis. CS gas can cause paresthesia, erythema, edematous dermatitis, and in severe cases, vesiculobullous eruptions, and partial thickness burns.^7^

This case represents a unique presentation of tear gas exposure precipitating disseminated herpes zoster in a patient receiving immunosuppressive therapy. To our knowledge, there are no reported cases describing disseminated herpes zoster following tear gas exposure. Our patient’s treatment with a JAK inhibitor, known to impair antiviral cell-mediated immunity, likely lowered the threshold for VZV reactivation following acute chemical-induced cutaneous inflammation. One potential mechanism is that CS gas–induced inflammation and physiologic stress lead to transient dysregulation of cell-mediated immunity, facilitating VZV reactivation and dissemination in the setting of immunosuppression. A case report of disseminated zoster in a patient with extensive burns suggests that burn-related immunosuppression and cytokine modulation may contribute, although reactivation of VZV is rare in burn patients and the precise mechanism remains unclear.^8^

VZV reactivation is linked to impaired cell-mediated immunity, whether due to physiologic stress or immunosuppression. Specifically, JAK inhibitors are associated with an increased risk of herpes zoster. Multiple studies, including a systematic review and meta-analysis, demonstrate higher rates of herpes zoster in patients receiving JAK inhibitors compared with placebo (1.56% vs 0.99%).^3^^,^^5^^,^^6^^,^^9^

JAK inhibitors disrupt Janus kinase - signal transducer and activator of transcription signaling, including interferon-γ, IL-2, and IL-15, leading to impaired CD4+ and CD8+ T-cell responses. While this provides therapeutic benefit to patients with inflammatory conditions, it alters interferon-mediated antiviral immunity. As a result, immune surveillance of latent viruses is reduced, leading to higher rates of VZV reactivation. Given this increased risk, patients should be counseled prior to initiating JAK inhibitor therapy, including upadacitinib, and recombinant zoster vaccination should be considered before treatment initiation. Current Centers for Disease Control and Prevention guidelines recommend a 2-dose series of the recombinant zoster vaccine for adults aged ≧ 19 years who are or will be immunodeficient or immunosuppressed, including those starting or receiving JAK inhibitors, preferably before initiation of therapy when feasible.^10^

Prompt recognition of disseminated VZV reactivation is critical, particularly in immunocompromised patients. Polymerase chain reaction remains the diagnostic gold standard and should be performed when vesicular, ulcerated, or crusted lesions are present. Early antiviral therapy is crucial because disseminated VZV can cause severe systemic and neurological complications, including meningoencephalitis, myelitis, and vasculopathy.^4^ Recognition, confirmation, and antiviral treatment are essential in preventing morbidity and potential mortality.

In conclusion, this case describes a novel presentation of tear gas–associated disseminated herpes zoster in a patient receiving JAK inhibitor therapy. It highlights the challenges that can arise when distinguishing chemical-induced eruptions from infectious etiologies. In immunomodulated patients, eruptions should prompt suspicion of viral reactivation and warrant diagnostic testing and early initiation of antiviral therapy to reduce morbidity.

## Conflicts of interest

None disclosed.
